# SARS coronavirus papain-like protease induces Egr-1-dependent up-regulation of TGF-β1 via ROS/p38 MAPK/STAT3 pathway

**DOI:** 10.1038/srep25754

**Published:** 2016-05-13

**Authors:** Shih-Wein Li, Ching-Ying Wang, Yu-Jen Jou, Tsuey-Ching Yang, Su-Hua Huang, Lei Wan, Ying-Ju Lin, Cheng-Wen Lin

**Affiliations:** 1 Department of Medical Laboratory Science and Biotechnology, China Medical University, Taichung, Taiwan; 2 Department of Biotechnology and Laboratory Science in Medicine, National Yang Ming University, Taipei, Taiwan; 3 Department of Biotechnology, Asia University, Wufeng, Taichung, Taiwan; 4 Department of Medical Genetics and Medical Research, China Medical University Hospital, Taichung, Taiwan

## Abstract

SARS coronavirus (SARS-CoV) papain-like protease (PLpro) has been identified in TGF-β1 up-regulation in human promonocytes (Proteomics 2012, 12: 3193-205). This study investigates the mechanisms of SARS-CoV PLpro-induced TGF-β1 promoter activation in human lung epithelial cells and mouse models. SARS-CoV PLpro dose- and time-dependently up-regulates TGF-β1 and vimentin in A549 cells. Dual luciferase reporter assays with TGF-β1 promoter plasmids indicated that TGF-β1 promoter region between −175 to −60, the Egr-1 binding site, was responsible for TGF-β1 promoter activation induced by SARS-CoV PLpro. Subcellular localization analysis of transcription factors showed PLpro triggering nuclear translocation of Egr-1, but not NF-κB and Sp-1. Meanwhile, Egr-1 silencing by siRNA significantly reduced PLpro-induced up-regulation of TGF-β1, TSP-1 and pro-fibrotic genes. Furthermore, the inhibitors for ROS (YCG063), p38 MAPK (SB203580), and STAT3 (Stattic) revealed ROS/p38 MAPK/STAT3 pathway involving in Egr-1 dependent activation of TGF-β1 promoter induced by PLpro. In a mouse model with a direct pulmonary injection, PLpro stimulated macrophage infiltration into lung, up-regulating Egr-1, TSP-1, TGF-β1 and vimentin expression in lung tissues. The results revealed that SARS-CoV PLpro significantly triggered Egr-1 dependent activation of TGF-β1 promoter via ROS/p38 MAPK/STAT3 pathway, correlating with up-regulation of pro-fibrotic responses *in vitro* and *in vivo*.

Severe acute respiratory syndrome (SARS)–associated coronavirus (SARS-CoV) is the causative agent of SARS outbreak in 2003^1^,^2^. SARS-CoV infection induces severe respiratory illnesses, such as bronchial epithelial denudation, loss of cilia, multinucleated syncytial cells, squamous metaplasia and transendothelial migration of monocytes/macrophages and neutrophils into lung tissue^3^,^4^. SARS-CoV triggers a pro-inflammatory cytokine storm that links with pulmonary fibrosis of SARS patients^5^,^6^. Near 20% of SARS patients recovered still have lung fibrosis 9 months post infection^7^,^8^.

SARS-CoV contains a single stranded and positive sense RNA genome that has a 5′ cap and a 3′ poly-(A) tract containing 14 open reading frames (ORFs)^1^,^2^. ORF1a and ORF1ab at the 5′ proximal end encode two large overlapping replicase polyproteins 1a and 1ab (~450 and ~750 kDa, respectively). Two specific embedded proteases, papain-like (PLpro) and 3C-like (3CLpro), mediate the proteolytic processing of polyproteins 1a and 1ab precursors into 16 nsps (termed nsp1–16). SARS-CoV PLpro is identified as a deubiquitinating enzyme that removes Lys48-linked polyubiquitin chains^9^. PLpro blocks polyI:C-induced activation of IRF3, NF-κB, and STAT1, correlating with the inhibitory effect on interferon (IFN) induction and signaling^10^. Recent, SARS-CoV PLpro exhibits the deubiquitinating enzymatic activity on RIG-I, STING, TRAF3, TBK1, and IRF3, in which suppresses STING-TRAF3-TBK1 signaling pathway, then negatively regulating IRF3 activation^11^. Our laboratory demonstrates type I IFN antagonist mechanism of SARS-CoV PLpro correlating with the activation of ubiquitin-proteosome pathway, in which inhibits type I IFN-induced phosphorylation of STAT1 Ser727 via the degradation of ERK1^12^. In addition, we also indicated that SARS-CoV PLpro significantly triggers the up-regulation of Transforming growth factor beta 1 (TGF-β1) and pro-fibrotic genes via ubiquitin proteasome, p38 MAPK, and ERK1/2-mediated signaling pathways in human promonocytes^13^. Therefore, PLpro modulates the innate immune response as well as involve in the pathogenesis of SARS-CoV-induced pulmonary fibrosis.

TGF-β1 rises in plasma and lung tissues in patients during the early phase of SARS^14^,^15^. TGF-β1 is the major mediator of fibrosis, up-regulating the expression of pro-fibrotic genes such as vimentin, type I and type III collagen^16^,^17^. Several transcription factors such as AP-1, Sp1, NF-κB, Egr-1, and STAT-3 transactivate TGF-β1 promoter, in which are regulated by various cellular kinases^18^. Latent TGF-β1 in the extracellular matrix is proteolytically cleaved by pro-protein convertases (furin, thrombospondin-1 (TSP-1), matrix metalloproteinase-2 (MMP-2) and MMP-9), then becomes as bioactive TGF-β1^19^,^20^,^21^. TGF-β1 activates Smad2/3, PI3K/Akt, ERK1/2, GSK-3β, and/or p38 MAPK signaling pathways for induction of tissue fibrosis, and epithelial-mesenchymal transition (EMT)^22^. Thus, this study will investigate the molecular mechanisms of PLpro-induced TGF-β1 promoter activation in human lung epithelial cells and mouse lung tissues, as well as elucidate the role of TGF-β1 promoter-specific transcription factors in SARS-related pulmonary pathogenesis.

## Materials and Methods

### Cell culture and transient transfection with pSARS-PLpro

Human alveolar basal epithelial A549 cells were grown in Dulbecco’s Modified Eagle’s Medium (DMEM; HyClone Laboratories, Logan, Utah, USA) with 100 U/mL of penicillin and streptomycin, 2 mM _L-_glutamine, and 10% fetal bovine serum (FBS; Biological Industries, Kibbutz Beit Haemek, Israel). SARS-CoV PLpro gene, nt 4507–5840 of the SARS-CoV TW1 strain (GenBank accession no. AY291451) was amplified by RT-PCR, and then cloned into expression vector pcDNA3.1/His C (Invitrogen), as described in our prior reports^12^,^13^. The empty vector pcDNA3.1 or pSARS-PLpro at the concentrations of 0, 0.5, 1, 2, 5, and 10 μg/ml was transfected into A549 cells with Arrest-In transfection reagent (Thermo scientific). After 5-h incubation, transfected cells were maintained in DMEM medium containing 20% FBS. Transient expression of recombinant PLpro in A549 cells 2 days post transfection was analyzed using immunefluoresce staining and Western blotting with mouse polyclonal antibodies against anti-*E. coli* synthesized PLpro, as described in our prior reports^12^,^13^.

### Quantifying relative mRNA expression of fibrotic genes using real-time RT-PCR

To measure the expression of SARS PLpro, TGF-β1, pro-fibrotic and pro-protein convertase genes in transfected cells or mouse lung tissues, total RNAs were extracted from transfected A549 cells with empty vector pcDNA3.1 or pSARS-PLpro 2 days post transfection using PureLink Micro-to-Midi Total RNA Purification System kit (Invitrogen). Relative mRNA levels were analyzed using two-step real time RT-PCR with SYBR Green I, as described in our prior reports^11^,^12^. Primer pairs of SARS PLpro, TGF-β1, pro-fibrotic and pro-protein convertase genes were listed in Table 1. Quantification of specific PCR products was performed using the ABI Prism 7900HT Sequence Detection System (PE Applied Biosystems). Relative changes in mRNA level of indicated genes were normalized relative to GAPDH mRNA.

### Immunofluorescence staining assay

For determining the expression of PLpro and TGF-β1 as well as nuclear translocalization of transcription factors, A549 cells transiently transfected with pSARS-PLpro or empty vector grew on the glass coverslip in 6-well at 37 °C. After 2 days incubation, transfected cells were fixed with 3.7% formaldehyde in phosphate buffered saline (PBS) for 1 h, blocked with 1% bovine serum albumin (BSA) in PBS for the other 1 h, and then incubated with specific primary antibodies at 4 °C overnight, including mouse anti-SARS PLpro, rabbit anti-TGF-β1 (Cell signaling), rabbit anti-NF-κB p65 (Abcam), rabbit anti-Sp-1, rabbit anti-Egr-1 (Cell Signaling), and rabbit anti-vimentin (GeneTex). Subsequently, cells were reacted with FITC-conjugated goat anti-mouse or rabbit IgG plus DAPI (4′,6-diamidino-2-phenylindole) in dark box for 2 h. After washing three times with PBS, photographs of stained cells were taken using the immunofluorescence microscopy (Olympus, BX50).

### Western blotting assay

To determine protein expression and phosphorylation status, transfected A549 cells with empty vector pcDNA3.1 or pSARS-PLpro were harvested 2 days after transfection. Western blotting of cell lysates was accomplished, as described in our prior reports^12^,^13^. Resulting blots were probed with primary antibodies, including mouse polyclonal anti-*E. coli* synthesized PLpro, rabbit anti-vimentin (GeneTex), rabbit anti-TGF-β1 (Cell signaling), anti-α-SMA (Santa Cruz Biotechnology), rabbit anti-Egr-1, anti-phospho Erk1/2 (Thr202/Tyr204), anti-phospho p38 MAPK (Thr180/Tyr182), anti-phospho STAT3 (Ser727) (Cell Signaling), and anti-β-actin mAb (Abcam). Immune complexes were detected using HRP-conjugated goat anti-mouse or anti-rabbit IgG antibodies, as well as enhanced chemiluminescent HRP substrate (Millipore).

### Dual-luciferase reporter assay of TGF-β1 promoter activation

To test the activation of TGF-β1 promoter by SARS-CoV PLpro, PLpro-expressing and empty vector control cells were co-transfected with TGF-β1 promoter firefly luciferase reporter plasmids and internal control Renilla luciferase reporter pRluc-C1, as we reported earlier^12^. Full-length of truncated forms of TGF-β1 promoter plasmids (phTG1 −1362/+11, phTG5 −453/+11, phTG6 −323/+11, phTG7 −175/+11, phTG7-4 −60/+11) were kindly provided by Dr. S. J. Kim (CHA University, Republic of Korea)^23^. Mutated TGF-β1 promoter plasmid phTG5(Sp-1 mut), also named as Sp1mut −216/−215, was a kind gift from Prof. C. Weigert (University of Tuebingen, Germany)^24^. The activity of firefly and Renilla luciferase was measured 1 day post transient transfection by dual Luciferase Reporter Assay System (Promega) and the Clarity™ Luminescence Microplate Reader (BioTek Instruments).

### Detecting intracellular reactive oxygen species (ROS) by flow cytometry

PLpro-expressing and empty vector control cells were harvested 1 day post transient transfection, then stained with 10 μM 2,7-dichlorodihydrofluorescein diacetate (DCFH-DA, Sigma) at 37 °C for 30 min in darkroom. Final, DCFH-DA was converted to the fluorescent form DCF by ROS, DCF fluorescence represented as an intracellular ROS level was measured using flow cytometry with excitation wavelength of 485 nm and emission wavelength of 530 nm (Becton Dickinson FACS Calibur), as described in our prior report^25^.

### Inhibitor treatment and gene silencing by siRNA

For inhibitor treatment, vector control and PLpro-expressing cells were incubated with SB-431542 (TGF-β1 receptor inhibitor) (Tocris Bioscience), YCG063 (ROS inhibitor), SB203580 (p38 MAPK inhibitor), PD98059 (ERK1/2 inhibitor) (Calbiochem), and Stattic (STAT3 inhibitor) (Axon Medchem) at indicated concentrations, and then harvested for Western blotting and real-time RT-PCR, as described above. For the silence of Egr-1 and Rac1, vector control and PLpro-expressing cells were transfected with non-targeting control or Egr-1 siRNA (SMARTpool siRNA) (Thermo Scientific), and then harvested 24 h post transfection for Western blotting and real-time RT-PCR.

### Immunohistochemistry and real-time PCR assays of lung tissues from mice injected with pSARS-PLpro into chest

The mouse mode with a direct chest injection (Protocol No. 101-194-N) was reviewed and approved by the Institutional Animal Care and Use Committee (IACUC) at China Medical University. The animal use protocols were performed in accordance with the approved guidelines. Approximately 100 μl of 3% sucrose in PBS containing 50 μg of pSARS-PLpro, empty vector or solvent alone were injected into a right chest of mouse using a 1-ml syringe with a 28-gage needle every 2 days. Each group of 5 eight-weeks-old BALB/c male mice was injected 15 times, and then sacrificed. The lung tissues of each mouse in indicated groups were collected for immunohistochemistry (IHC) staining, and SYBR Green real time RT-PCR, respectively. For IHC staining, mouse lung tissues were fixed in formaldehyde and dehydrated in 70% ethanol for 30 min, in 95% ethanol for 30 min, and finally in 100% ethanol for 30 min. The tissues were embedded in paraffin at 58 °C, then cut at 4–15 μm thick section using a rotary microtome. Before staining, the sections were floated in a 56 °C water bath and mounted the sections onto slides. The slides with paraffin embedded section of mouse lung tissue were dewaxed in xylene 2 times for 5 min, rehydrated in 100% ethanol for 1 min, in 90% ethanol for 1 min, and finally in 80% ethanol for 1 min. Slides with mouse lung tissues were incubated in 3%H_2_O_2_ for 1 min to remove endogenous peroxidase activity, washed with PBS, and heated with 100 °C EDTA pH 9.0 for 20 min to induce antigen retrieval. Subsequently, slides were blocked with a protein block solution, and incubated with primary antibodies for 30 min, including mouse anti-*E. coli* synthesized PLpro serum, anti-mouse CD11b, anti-mouse TGFβ1 (Cell signaling), and anti-mouse vimentin (GeneTex). After washing with PBS, slides were reacted with Polymer-HRP for 20 min, developed using DAB substrate, and counterstained with hematoxylin. For collagen determination, the tissue sections were stained with Sirius red solution for 2 h, and then rinsed 10 times with 0.5% glacial acetic acid in PBS. After dehydrating with ethanol, stained sections were mounted on the glass slides, and then examined using light microscopy (Olympus, BX50). For quantitating relative mRNA levels, mouse tissue was homogenized, and performed as above described. Primer pairs of mouse TGF-β1 and pro-fibrotic genes were listed in Table 1.

### Statistical analysis

All data were calculated from 3 independent experiments. Student’s t-test or χ^2^ test was used to analyze all data. Statistical significance was considered at p < 0.05.

## Result

### SARS-CoV PLpro induced TGF-β1 production in human lung epithelial cells

**Figure 1:**
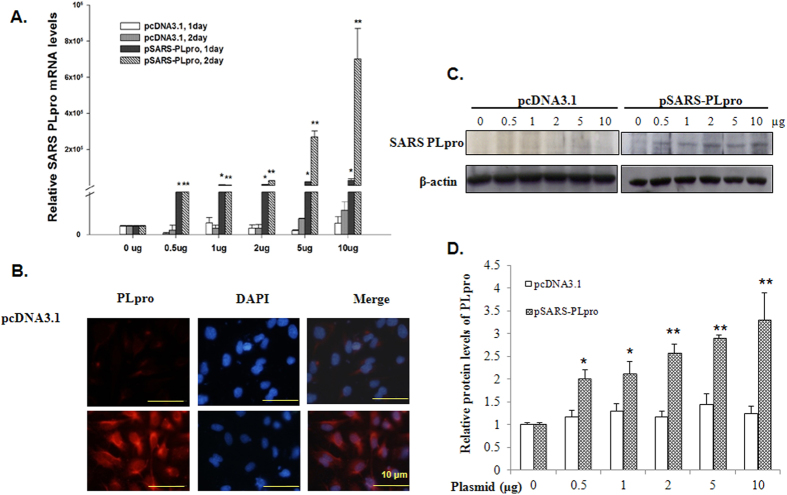
Expression of SARS-CoV PLpro in human alveolar basal epithelial cell line A549. A549 cells were transiently transfected with control vector or pSARS-CoV-PLpro. (**A**) The relative fold levels of SARS-CoV PLpro mRNA level are measured by quantitative real time PCR and normalized by GAPDH mRNA, presented as the relative ratio 1–2 days post transfection. (**B**)The SARS PLpro protein was detected using immunofluorescence staining of mouse polyclonal anti-SARS-CoV PLpro serum, rhodamine-conjugated anti-mouse IgG antibody and examined by fluorescence microscopy. (**C**) Lysates from cells transfected with pcDNA3.1 or pSARS-CoV PLpro were analyzed by 10% SDS-PAGE prior to blotting. The resulting blot was probed with the mouse polyclonal anti-SARS-CoV PLpro or anti-β actin antibody as an internal control. (**D**) Relative band intensity of PLpro was normalized by β actin, compared to the mock cell group, and quantified using imageJ based on triplicate replicates of each experiment. *p value < 0.05; **p value < 0.01 compared with vector control cells.

**Figure 2:**
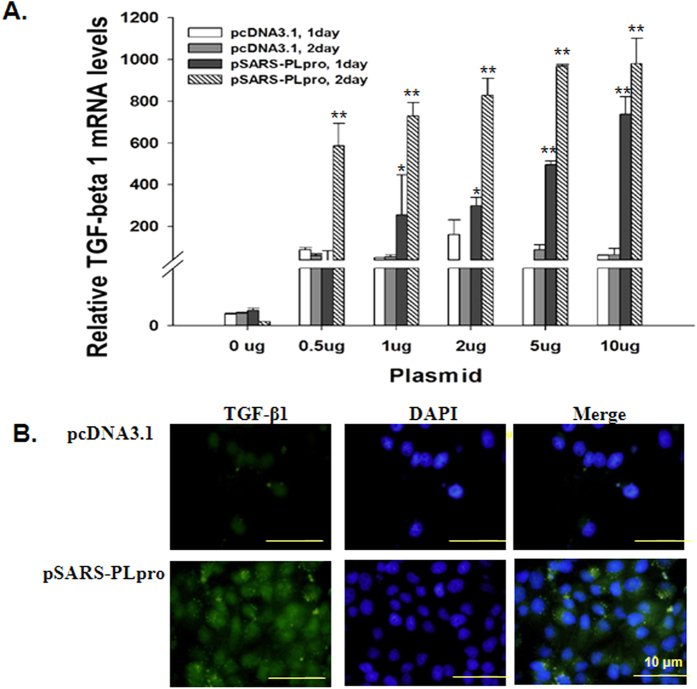
Relative mRNA and protein levels of TGF-β1 in transfected cells with vector control and pSARS-PLpro. (**A**) Total RNAs were extracted from transiently transfected cells with 10 μg of pcDNA3.1 and pSARS-PLpro 1–2 days after transfection. Relative TGF-β1 mRNA level was measured by quantitative real-time PCR, normalized by GAPDH mRNA, and then presented as the relative ratio. (**B**) The TGF-β1 protein was detected using immunofluorescence staining and examined by fluorescence microscopy. *p value < 0.05; **p value < 0.01 compared with vector control cells.

Our prior study demonstrated SARS-CoV PLpro triggering the TGFβ1 production in human promonocytes^13^, whether SARS PLpro induced TGF-β1 production in human lung epithelial A549 cells was further examined. Transient transfection of A549 cells with empty vector or pSARS-PLpro was performed to analyze the TGF-β1 production induced by SARS PLpro. Quantitative PCR, immunofluorescence staining, and Western blotting indicated transfection with pSARS-PLpro increasing the mRNA and protein levels of PLpro in A549 cells in a concentration-dependent manner, but not empty control vector (Fig. 1A–D). Meanwhile, relative mRNA levels of TGF-β1 in A549 cells were time- and concentration-dependently elevated following the transient transfection with pSARS-PLpro, but not vector control (Fig. 2A). Immunofluorescence staining assays indicated the protein levels of TGF-β1 obviously heightening in transfected cells with pSARS-PLpro compared to vector control (Fig. 2B). For examining the TGF-β1 induction of SARS-CoV PLpro in different cell lines, Huh7 (human hepatocarcinoma), H1299 (human non-small cell lung carcinoma), and ca9-22 (human oral cancer) cells were also evaluated (Supplemental Fig. 1). Real-time RT PCR analysis of transfected cells with pSARS-PLpro indicated that a lower level of TGF-β1 mRNA was detected in transfected H1299 cells compared to transfected A549 cells, but no significant level was found in transfected Huh7 and ca9-22 cells. In addition, comparison of the expression levels of PLpro and TGF-β1 among transfected cells with empty vector, pSARS-PLpro, and pBAC-SARSCoVΔES (a non-infectious SARS-CoV replicon) was further performed (Supplemental Fig. 2). The expression level of PLpro in transfected cells with pSARS-PLpro was 25-fold higher than the cells transfected with pBAC-SARSCoVΔES. A dose-dependent increase of TGF-β1 mRNA levels in A549 cells was induced by pSARS-PLpro and pBAC-SARSCoVΔES, respectively. Meanwhile, recombinant plasmids containing PLpro genes of MERS-CoV and HCoV NL63 (pMERS-PLpro, and pNL63-PLpro) were used for testing the specificity on the TGF-β1 induction compared to pSARS-PLpro (Supplemental Fig. 3). Interestingly, only SARS-CoV PLpro, but not ERS-CoV and HCoV NL63 PLpro, dose-dependently up-regulated the mRNA expression of TGF-β1. Therefore, the result demonstrated that SARS-CoV PLpro plays an important role in triggering a significant increase of TGF-β1 mRNA and protein levels in human lung epithelial cells.

### TGF-β1-dependent up-regulation of pro-fibrotic genes by PLpro

**Figure 3:**
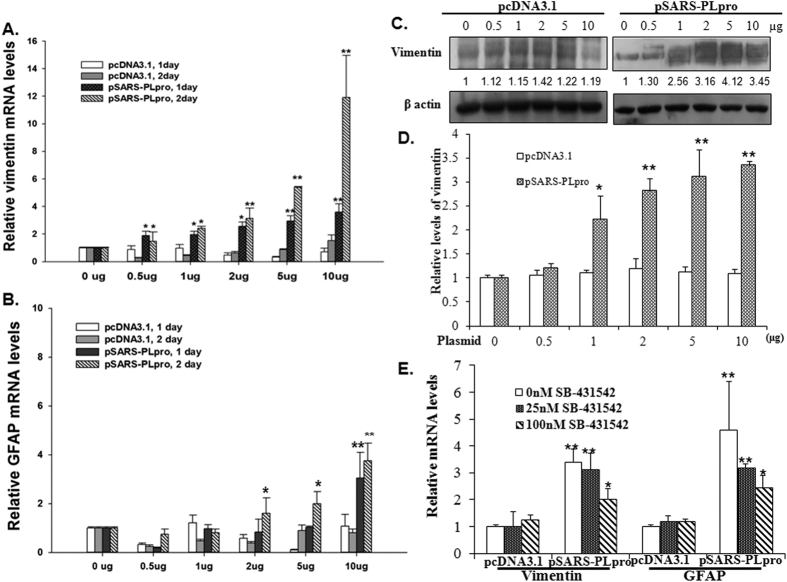
Analysis of vimentin and GFAP expression levels in transiently transfected cells. Relative mRNA levels of vimentin (**A**) and GFAP (**B**) were measured by quantitative real-time PCR 1–2 days post transfection with 10 μg of pcDNA3.1 and pSARS-PLpro, normalized by GAPDH mRNA, and presented as the relative ratio. The cell lysates were detected by Western blot and probed with anti-vimentin and anti-β actin antibodies as an internal control (**C**). Relative band intensity of vimentin was normalized by β actin, compared to the mock cell group, and quantified using imageJ based on triplicate replicates of each experiment (**D**). In addition, transfected cells were treated with an inhibitor of TGF-β1 receptor (SB-431542) 2 days post transfection, and harvested for measuring relative mRNA levels of vimentin and GFAP 1 day after treatment (**E**). *p value < 0.05; **p value < 0.01 compared with vector control cells.

To evaluate the correlation between TGF-β1 production and pro-fibrotic gene expression in PLpro-expressing cells, mRNA and protein levels of pro-fibrotic genes such as vimentin and glial fibrillary acidic protein (GFAP) in transfected cells were assessed using quantitative RT-PCR and Western blotting (Fig. 3). The mRNA levels of vimentin and GFAP were up-regulated in transfected cells with pSARS-PLpro, but not vector control (Fig. 3A,B). Besides, Western blotting showed the plasmid dose-dependent increase of vimentin proteins in PLpro-expressing cells, but not in vector controls (Fig. 3C,D). Next, a selective inhibitor of TGF-β1 receptor (SB-431542) was used to test the association of pro-fibrotic gene up-regulation with the TGF-β1 induction in PLpro-expressing cells (Fig. 3E). SB-431542 exhibited a dose-dependently inhibitory effect on SARS-PLpro-induced expression of vimentin (Fig. 3D). The result demonstrated SARS-CoV PLpro initiating TGF-β1-dependent up-regulation of pro-fibrotic genes in human lung epithelial cells.

### Egr-1-mediated activation of the TGF-β1 promoter induced by PLpro

**Figure 4:**
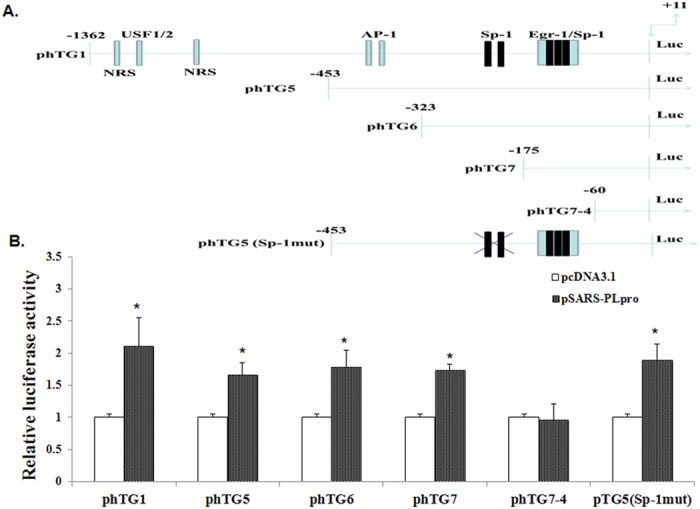
Activation of TGF-β1 promoter induced by PLpro. Wild type (phTG1), various deletion (phTG5, phTG6, phTG7, phTG7-4) and mutant (pTG5(Sp-1mut)) constructs of TGF-β1 promoter luciferase reporter were shown (**A**). A549 cells transfected with 10 μg of pcDNA3.1 and pSARS-PLpro were subsequently co-transfected with dual-luciferase reporters, and harvested for dual-luciferase reporter assays 1 day post transfection. TGF-β1 promoter-driven firefly luciferase and renilla luciferase were measured, and firefly luciferase activity normalized to renilla luciferase activity is reported (**B**). *p value < 0.05 compared with vector control cells.

To further examine the mechanism of TGF-β1 promoter activation induced by PLpro, wild type (phTG1), deletion (phTG5, phTG6, phTG7, phTG7-4), or mutant (phTG5(Sp1mut)) variants of TGF-β1 promoter-firefly luciferase reporters were used to examine the critical region for activation of TGF-β1 promoter in PLpro-expressing cells (Fig. 4A). In dual luciferase reporter assays, vector and PLpro-expressing cells were co-transfected with an internal control Renilla luciferase reporter and indicated TGF-β1 promoter-firefly luciferase reporter. Dual luciferase reporter assays indicated greater than 1.8-fold increases of firefly leucuferase activity by phTG1, phTG5, phTG6, phTG7, and phTG5(Sp1mut) respectively in PLpro-expressing cells than vector control cells (Fig. 4B). However, PLpro did not trigger the activation of TGF-β1 promoter deletion form phTG7-4. The result indicated the promoter region between −175 to −60, the Egr-1/Sp-1 binding site, as responsible for PLpro-induced activation of TGF-β1 promoter-firefly luciferase.

**Figure 5:**
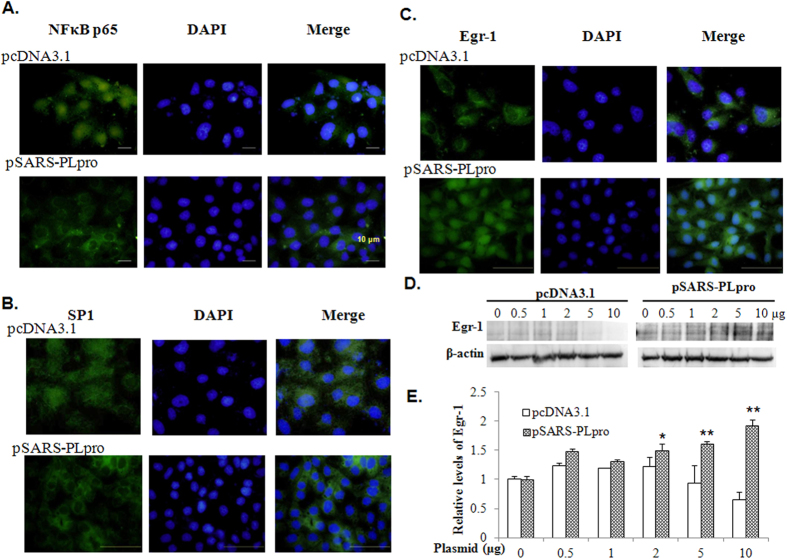
Effect of PLpro on nuclear translocation and expression of NF-κB, Sp-1 and Egr-1 in A549 cells. For analyzing subcellular location of NF-κB (**A**), Sp-1 (**B**) and Egr-1 (**C**), A549 cells transfected with 10 μg of pcDNA3.1 or pSARS-PLpro were washed, fixed, and reacted with indicated primary antibodies and FITC-conjugated secondary antibodies. Finally, cells were stained with DAPI for 10 minutes, imaging analyzed by immunofluorescent microscopy. Meanwhile, cell lysates of transiently transfected cells were detected by Western blot and probed with anti-Egr-1 or anti-β-actin antibody as an internal control (**D**). Relative band intensity of Egr-1 was normalized by β actin, compared to the mock cell group, and quantified using imageJ based on triplicate replicates of each experiment (**E**). *p value < 0.05; **p value < 0.01 compared with vector control cells.

**Figure 6:**
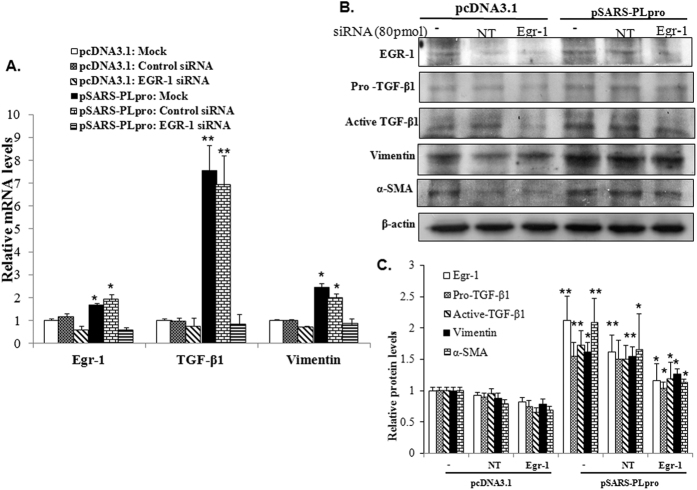
Functional analysis of Egr-1 with siRNA-mediated gene silencing. A549 cells transfected with 10 μg of pcDNA3.1 or pSARS-PLpro were re-transfected with negative control or Egr-1siRNA were harvested 1 day post transfection for quantitative PCR (**A**) and Western blotting (**B**) assays. Relative mRNA levels of Egr-1, TGF-β1, and vimentin were normalized by GAPDH mRNA, presenting as relative ratio. The Western blot was probed with indicated primary antibodies as an internal control, detected using HRP-conjugated secondary antibodies and chemiluminescent HRP substrate. Relative band intensity of indicated proteins was normalized by β actin, compared to the mock cell group, and quantified using imageJ based on triplicate replicates of each experiment (**C**). *p value < 0.05; **p value < 0.01 compared with vector control cells.

To further investigate the nuclear localization of NF-κB, Sp-1 and Egr-1, both types of cells were analyzed by immunofluorescence staining with primary and FITC-conjugated secondary antibodies, plus DAPI nuclear counterstain (Fig. 5). Immunofluorescence imaging analysis indicated NF-κB and Sp-1 were localized in the nucleus as well as Erg-1 was localized in the cytoplasm of vector control cells (Fig. 5A–C). However, PLpro stimulated the translocation of Erg-1 into the nucleus, but inactivated the NF-κB and Sp-1 that were localized in the cytoplasm as an inactive complex (Fig. 5A–C). The finding correlated with the previous report in that SARS CoV PLpro has the inhibitory ability on the activation of NF-κB into nucleus^10^. In addition, Western blotting indicated SARS-CoV PLpro causing the increased expression of Egr-1 in plasmid dose-dependent manners (Fig. 5D,E). Subsequently, gene silencing of Erg-1 by RNA interference was performed to examine the role of Egr-1 in PLpro-induced up-regulation of TGF-β1 and pro-fibrotic responses (Fig. 6). Egr-1 siRNA, not non-targeting siRNA, definitely reduced mRNA and protein levels of Egr-1, TGF-β1, vimentin and α-SMA in PLpro-expressing cells, but slightly decreased them in vector control cells. The result demonstrated gene silencing of Egr1 linked with the decrease of TGF-β1 promoter activation and pro-fibrotic responses in PLpro-expressing cells. The finding revealed Egr-1 up-regulated by SARS-CoV PLpro playing a crucial role in the activation of TGF-β1 promoter, as well as the induction of TGF-β1-mediated pro-fibrotic responses in SARS pathogenesis.

### PLpro-induced Egr-1dependnent up-regulation of thrombospondin-1

**Figure 7:**
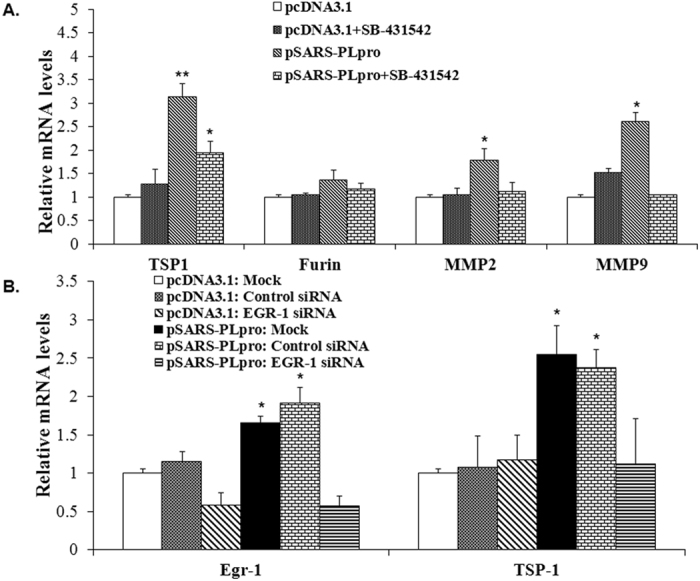
Effect of TGF-β1 receptor inhibitor and silencing Egr-1 on PLpro-induced mRNA expressin of pro-protein convertase. A549 cells transfected with 10 μg of pcDNA3.1 or pSARS-PLpro were treated with TGF-β1 receptor inhibitor (SB-431542) (**A**), or transfected with negative control or Egr-1siRNA (**B**), and then harvested 1 day post treatment or transfection for measuring relative mRNA levels of indicated pro-protein convertases. *p value < 0.05; **p value < 0.01 compared with vector control cells.

To examine the expression of pro-protein convertases, relative mRNA levels of thrombospondin-1 (TSP-1), furin, matrix metallopeptidase 2 (MMP2) and MMP9 were measured in vector control and PLpro-expressing cells (Fig. 7). Quantitative PCR analysis indicated that TSP-1, MMP-2 and MMP-9 were up-regulated in PLpro-expressing cells versus vector control (Fig. 7A). Interestingly, the inhibitor of TGF-β1 receptor (SB-431542) significantly reduced PLpro-induced expression of MMP-2 and MMP-9 at baseline, but partially inhibited the expression of TSP-1 (Fig. 7A). The finding implied bioactive TGF-β1 induced by PLpro directly stimulated the expression of MMP2 and MMP9, and also associated with the TSP-1 up-regulation. In addition, the correlation between the expression levels of Egr-1 and TSP-1 induced by PLpro was further examined (Fig. 7B). Egr-1 gene silencing significantly reduced the mRNA expression of TSP-1 in PLpro-expressing cells (Fig. 7B). The results indicated that Egr-1-dependent up-regulation of TSP-1 as well as TGF-β1-mediated activation of MMP2, MMP9, and TSP-1 expressions were responsible for the proteolytic cleavage of latent TGF-β in PLpro-expressing cells.

### ROS/p38 MAPK/STAT3 pathway was responsible for PLpro-induced Egr-1 dependent TGF-β1-mediated pro-fibrosis

**Figure 8:**
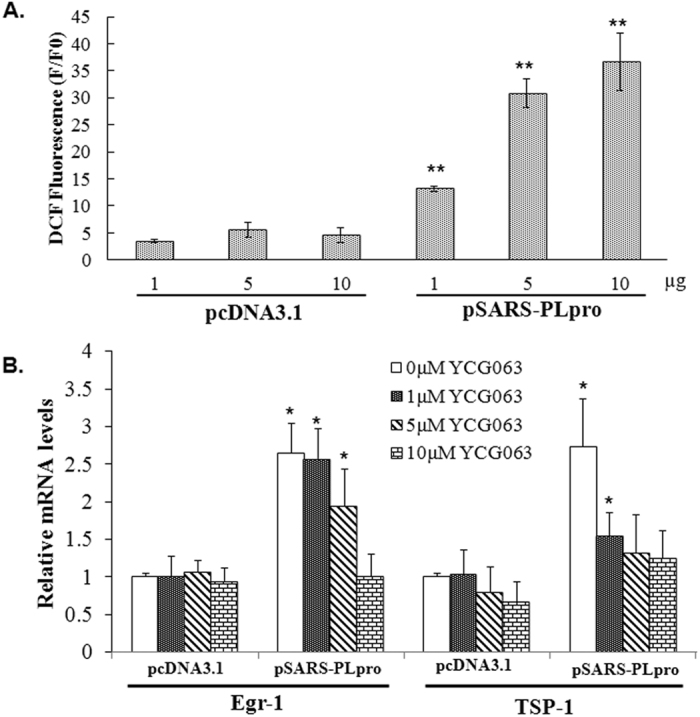
Functional analysis of intracellular ROS in PLpro-induced Egr-1 up-regulation. For flow cytometric analysis of intracellular ROS levels, A549 cells transfected with 10 μg of pcDNA3.1 or pSARS-PLpro were stained using DCFH-DA, and then measured by flow cytometry. Relative changes of intracellular ROS levels were shown (**A**). For analyzing the effect of ROS on the Egr-1 expression, both types of cells were treated with ROS inhibitor (YCG063), and then harvested for quantitating relative changes of Egr-1 and TSP-1 mRNA, in which was normalized by GAPDH mRNA and presented as the relative ratio (**B**). *p value < 0.05; **p value < 0.01 compared with vector control cells.

**Figure 9:**
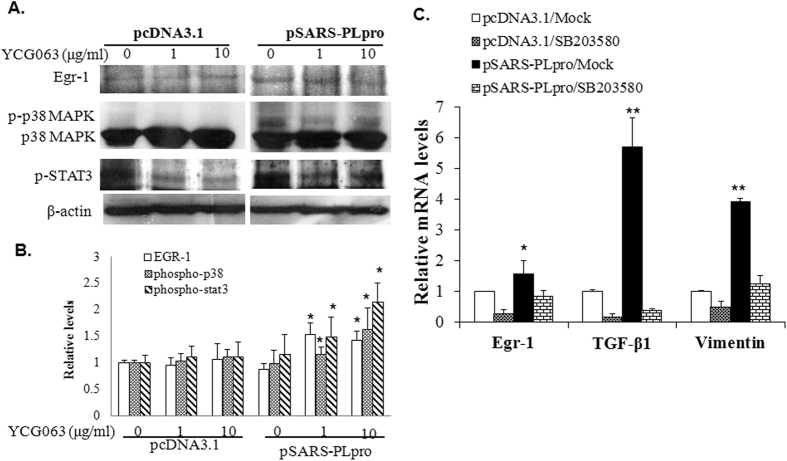
Analysis of p38 MAPK activity in ROS-mediated Egr-1 up-regulation induced by PLpro. For analysis of ROS-mediated signaling, A549 cells transfected with 10 μg of pcDNA3.1 or pSARS-PLpro were treated with or without ROS inhibitor (YCG063), and then harvested for Western blotting assays with specific primary antibodies against Egr-1, phospho-p38 MAPK, and phospho-STAT3 (**A**). Relative band intensity of indicated proteins was normalized by β actin, compared to the mock cell group, and quantified using imageJ based on triplicate replicates of each experiment (**B**). For conforming the role of p38 MAPK in ROS-dependent Egr-1 up-regulation, both types of cells were treated with p38 MAPK inhibitor (SB203580), and then harvested for quantitating relative changes of Egr-1, TGF-β1, and vimentin mRNA, in which was normalized by GAPDH mRNA and presented as the relative ratio (**C**). *p value < 0.05; **p value < 0.01 compared with untreated cells.

**Figure 10:**
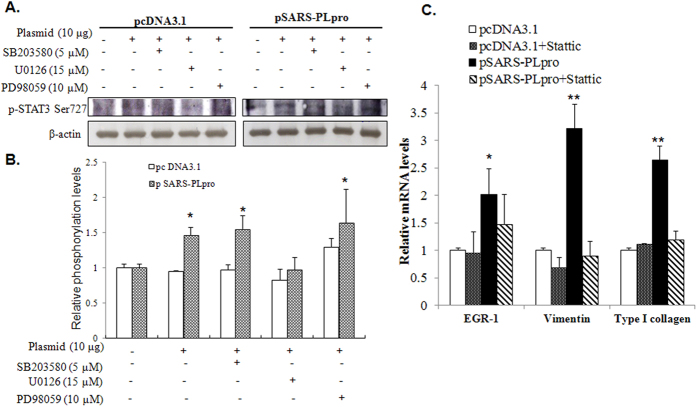
Analysis of STAT3 activity in p38 MAPK-mediated pathway of PLpro-induced Egr-1 up-regulation. For analysis of p38 MAPK-dependent signaling, A549 cells transfected with 10 μg of pcDNA3.1 or pSARS-PLpro were treated with or without p38 MAPK, MEK, and ERK inhibitors (SB203580, U0126, and PD98059), and then harvested for Western blotting assays with specific primary antibodies against phospho-STAT3 (**A**). Relative band intensity of indicated proteins was normalized by β actin, compared to the mock cell group, and quantified using imageJ based on triplicate replicates of each experiment (**B**). For conforming the role of STAT3 in p38 MAPK-dependent Egr-1 up-regulation, both types of cells were treated with STAT3 inhibitor (Stattic), and then harvested for quantitating relative changes of Egr-1, vimentin, and type I collagen mRNA, in which was normalized by GAPDH mRNA and presented as the relative ratio (**C**). *p value < 0.05; **p value < 0.01 compared with vector control cells.

Since the intracellular ROS generation was reported to modulate the expression of Egr-1 and TSP-1^26^,^27^, the involvement of ROS-mediated pathway in PLpro-induced Egr-1-depedent activation of TGF-β1 and pro-fibrotic responses was explored (Figs 8, 9, 10). Intracellular ROS levels in PLpro-expressing and vector control cells were detected using flow cytometry with DCFH-DA staining (Fig. 8A). DCF fluorescence analysis indicated PLpro triggered the ROS generation in a dose dependent manner. Meanwhile, ROS inhibitor (YCG063) concentration-dependently reduced PLpro-induced up-regulation of Egr-1 and TSP-1(Fig. 8B). For insight into the pathway of ROS-mediated Egr-1 up-regulation, the activity of MAP kinases and transcription factors were analyzed using Western blotting (Fig. 9A,B). ROS inhibitor (YCG063) significantly reduced PLpro-induced phosphorylation of p38 MAPK and STAT3. Furthermore, p38 MAPK inhibitor (SB203580) notably declined the PLpro-induced expression of Egr-1, TGF-β1 and vimentin, as well as PLpro-induced phosphorylation of STAT3 (Figs 9C and 10A,B). STAT3 inhibitor (Stattic) also diminished PLpro-induced expression of Egr-1, vimentin, and Type I collagen (Fig. 10C). Therefore, results showed SARS-CoV PLpro up-regulating Egr-1 dependent TGF-β1 mediated pro-fibrosis via ROS/p38 MAPK/STAT3 pathway.

### Pulmonary pro-fibrotic activity of SARS-CoV PLpro in a mouse model

**Figure 11:**
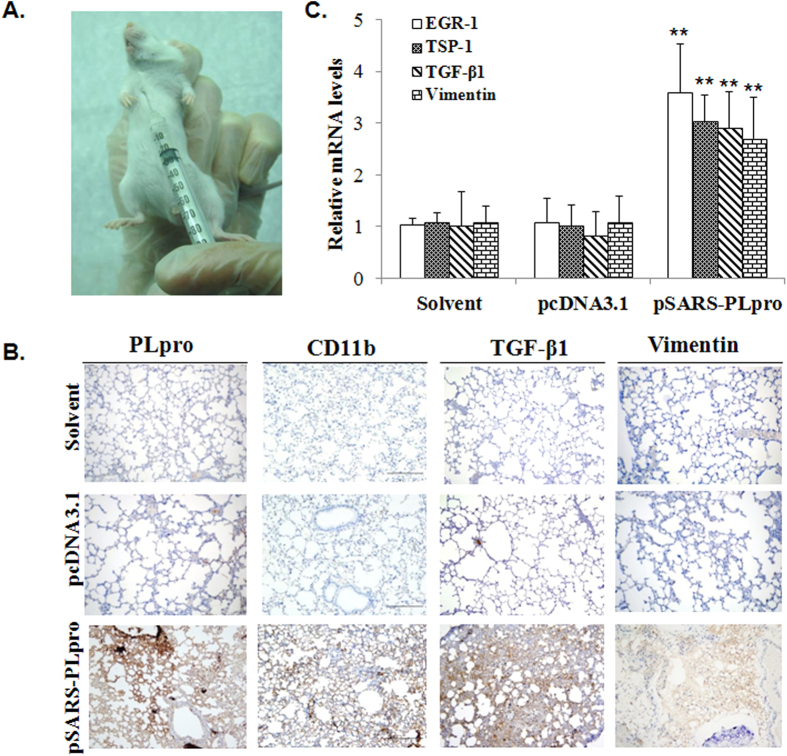
A mouse model of SARS-CoV PLpro-induced pulmonary pro-fibrosis. (**A**) The chest of BALB/c mice was directly injected with solvent, 10 μg of pcDNA3.1, or pSARS-PLpro every 2 days. (**B**) The lung tissues were collected after 15-times of chest injection, embedded, and sectioned. The lung tissue sections were performed using IHC staining with anti-PLpro sera, anti-CD11b, anti-TGF-β1, and anti-vimentin antibodies. (**C**) Total RNAs of the lung tissues were extracted; relative mRNA levels of Egr-1, TSP-1, TGF-β1, and vimentin were quantitated using real-time PCR, normalized by GAPDH mRNA, and presented as the relative ratio. *p value < 0.05; **p value < 0.01 compared with the solvent control group.

A mouse model that direct injected with empty vector or pSARS-PLpro into the mouse lung was set up for examining the pro-fibrotic activity of PLpro *in vivo* (Fig. 11). The mice were injected 15 times with indicated plasmids into the chest every two days (Fig. 11A), and then sacrificed and collected the lung tissue for tissue immunohistochemistry stain and quantitative RT-PCR (Fig. 11B,C). Immunohistochemistry staining with anti-PLpro immunized sera indicated a significant expression of SARS-PLpro in lung tissues of mice injected with pSARS-PLpro, but not empty vector or solvent control (Fig. 11B). Lung infiltration of immune cells, particular CD11b monocytes, was identified in pulmonary alveoli expressing SARS-PLpro, but not the controls, using immunohistochemistry staining with anti-mouse CD11b mAb. In addition, PLpro, but not the controls triggered a significant increase of TGF-β1 and vimentin protein levels in the lung tissues. Subsequently, real-time PCR confirmed PLpro raised the mRNA expression of Egr-1, TSP-1, TGF-β1, and vimentin in mouse lung tissues versus empty vector or solvent control (Fig. 11C). Overall, result of the *in vivo* experiments was in accord with the *in vitro* data that SARS PLpro substantially stimulated Egr-1 dependent TGF-β1 mediated pro-fibrotic responses.

## Discussion

This study verified SARS-CoV PLpro inducing TGF-β1 mediated pro-fibrotic responses in human lung epithelial cells and mouse lung tissues (Figs 1, 2, 3 and 11), according with the previous report in that PLpro up-regulated TGF-β1 and its associated genes such as glial fibrillary acidic protein (GFAP) and vimentin^13^. Except SARS-CoV nucleocapsid^28^, PLpro was identified to generate the TGF-β1 production that linked to activate the pro-fibrotic responses. Among SARS-CoV-induced cytokines^5^,^6^, TGF-β1 could be associated with the induction of lung fibrosis. Therefore, SARS-CoV PLpro plays an important role in the TGF-β1-mediated pulmonary fibrosis of SARS pathogenesis.

This study proved SARS-CoV-PLpro heightening the role of Egr-1 in TGF-β1-mediated pro-fibrosis, in which increased the expression and nuclear translocalization of Egr-1, as well as strengthened the transcription of Egr-1-responsive genes (TGF-β1 and TSP-1) (Figs 5, 6, 7). In addition, gene silencing by siRNA confirmed the importance of Egr-1 on TGF-β1-mediated pro-fibrosis induced by PLpro (Fig. 6). Previous studies demonstrated Egr-1 exhibiting potent stimulatory action on fibrotic gene expression, and correlating with human fibrotic disorders like emphysema, pulmonary fibrosis, and systemic sclerosis^29^,^30^. Those results revealed TGF-β increasing Egr-1 protein and mRNA levels, stimulating Egr-1-depdent transcription of collagen, and then triggering Smad-independent fibrotic response. However, our study indicated SARS-CoV PLpro causing Egr-1-depdent transcription of TGF-β1, in which was associated with TGF-β1-mediated up-regulation of pro-fibrotic genes (such as vimentin, GFAP, and α-SMA). Of pro-protein convertases^19^,^20^,^21^, Egr-1 dependent up-regulation of TSP-1 in PLpro-expressing cells was discovered (Figs 7B and 8B), in which TSP-1 was suggested as responsible for the proteolytic cleavage of latent TGF-β1. The finding directed the alternative role of Egr-1 in TGF-β1 induction and TSP-1-mediated latent TGF-β1 activation in PLpro-expressing cells versus vector controls. Therefore, Egr-1 plays an important mediator in SARS CoV PLpro-induced pro-fibrotic response, appearing as a potential target for anti-fibrotic therapies in SARS patients.

**Figure 12:**
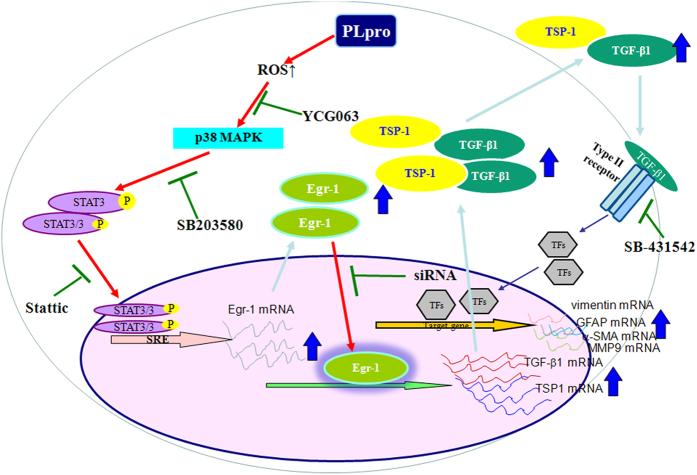
The proposed model for the mechanism of Egr-1-dependent TGF-β1-mediated pulmonary pro-fibrosis induced by SARS-CoV PLpro via ROS/p38 MAPK/STAT3 pathway.

The study identified ROS/p38 MAPK/STAT3/Egr-1 pathway involving in PLpro-induced TGF-β1-mediated pro-fibrotic response (Fig. 12). The unique pathway has been confirmed using specific inhibitors of ROS (YCG063), p38 MAPK (SB203580) and STAT3 (Stattic) (Figs 8, 9, 10), as in accord with our previous report in that SARS-CoV PLpro triggered TGF-β1 production via p38 MAPK mediated signaling in human promonocytes^13^. In addition, ROS was associated with the up-regulation and activation of Egr-1 in hypoxia induced pulmonary fibrosis, hypertension, apoptosis, and atherosclerosis^26^,^31^. The Egr-1 promoter consists of several functional response elements (such as serum response element (SRE), cAMP response element (CREs), APETALA1 (AP1), gene-specific activator protein 1 (Sp1), and Ets-family transcription factor binding sites)^31^. Several pathways of ROS-mediated Egr-1 up-regulation were identified, including MEK/ERK, c-Jun N-terminal kinases (JNKs), ERK-1/2-Elk-1 and NF-kappaB^31^,^32^. The increase of ROS production generally activated MAPK pathways (ERKs, JNKs, or p38 MAPKs) in response to growth factors, cytokines, and various stresses^33^. In addition, the interaction between MAPK and STAT3 has been demonstrated in lipopolysaccharide-triggered inflammatory lung diseases^34^. Therefore, ROS/p38 MAPK/STAT3 was reasonably responsible for the Egr-1 upregulation of TGF-β1-mediated pro-fibrotic response induced by SARS-CoV PLpro.

In conclusion, SARS-CoV PLpro significantly induced the TGF-β1-mediated pro-fibrotic response via ROS/p38 MAPK/STAT3/Egr-1 pathway *in vitro* and *in vivo*. PLpro also triggered Egr-1 dependent transcription of TSP-1 as an important role in latent TGF-β1 activation. Therefore, Egr-1 plays a critical mediator in SARS-CoV PLpro induced pathogenesis, as a potential therapeutic target to prevent the pulmonary fibrosis induced by SARS-CoV.

## Additional Information

**How to cite this article**: Li, S.-W. *et al.* SARS coronavirus papain-like protease induces Egr-1-dependent up-regulation of TGF-β1 via ROS/p38 MAPK/STAT3 pathway. *Sci. Rep.* **6**, 25754; doi: 10.1038/srep25754 (2016).

## Supplementary Material

Supplementary Information

## Figures and Tables

**Table 1:** Primer pairs for real time PCR used in the study.

Gene name	Forward primer	Reverse primer
SARS-CoV PLpro	5′-GTCAAATTCAATGCACCAGCAC-3′	5′-ATCACCAAGCTCGCCAACAG-3′
Human TGF-β1	5′-GGCCTTTCCTGCTTCTCATGG-3′	5′-CCTTGCTGTACTGCGTGTCC-3′
Human vimentin	5′-TCTCTGAGGCTGCCAACCG-3′	5′-CGAAGGTGACGAGCCATTTCC-3′
Human GFAP	5′-GAGCAGGAGGAGCGGCAC-3′	5′-TAGGTGGCGATCTCGATGTCC-3′
Human Type I collagen	5′-GTTCGTGACCGTGACCTCG-3′	5′-TCTTGTCCTTGGGGTTCTTGC-3′
Human Egr-1	5′-CTCTCCAGCCTGCTCGTC-3′	5′-AGCAGCATCATCTCCTCCAG-3′
Human TSP-1	5′-CACCAACCGCATTCCAGAG-3′	5′-TCAGGGATGCCAGAAGGAG-3′
Human furin	5′-CCGCAGATGGGTTTAATGAC-3′	5′-TTGTTGGCTTCGCTGGTG-3′
Human MMP2	5′-CCCTGATGTCCAGCGAGTG-3′	5′-ACGACGGCATCCAGGTTATC-3′
Human MMP9	5′-CCAACCACCACCACACCG-3′	5′-GCAGGCATCGTCCACCG-3′
Human IL-17	5′-CGCAATGAGGACCCTGAGAG-3′	5′-GCCCACGGACACCAGTATC-3′
Human GAPDH	5′-AGCCACATCGCTCAGACAC-3′	5′-GCCCAATACGACCAAATCC-3′
Mouse TGF-β1	5′-CAACAATTCCTGGCGTTACCTTGG-3′	5′-GAAAGCCCTGTATTCCGTCTCCTT-3′
Mouse vimentin	5′-CTGTACGAGGAGGAGATGCG-3′	5′-AATTTCTTCCTGCAAGGATT-3′
Mouse TSP-1	5′- TGACCCTGGACTTGCTGTAG-3′	5′-CGGCTGCTGGACTGGTAG-3′
Mouse Egr-1	5′-GGAGCAAAGCCAAGCAAAC-3′	5′-ACGGAACAACACTCTGACAC-3′
Mouse GAPDH	5′-TGAGGCCGGTGCTGAGTATGTCG-3′	5′-CCACAGTCTTCTGGGTGGCAGTG-3′

## References

[b1] Marra M. A. *et al.* The Genome sequence of the SARS-associated coronavirus. Science 300, 1399–1404 (2003). 12730501 10.1126/science.1085953

[b2] Rota P. A. *et al.* Characterization of a novel coronavirus associated with severe acute respiratory syndrome. Science 300, 1394–1399 (2003). 12730500 10.1126/science.1085952

[b3] Nicholls J., Dong X. P., Jiang G. & Peiris M. SARS: clinical virology and pathogenesis. Respirology 8 Suppl, S6–8 (2003). 15018126 10.1046/j.1440-1843.2003.00517.x PMC7169081

[b4] Wang J. T. & Chang S. C. Severe acute respiratory syndrome. Current opinion in infectious diseases 17, 143–148 (2004). 15021055 10.1097/00001432-200404000-00013

[b5] Wong C. K. *et al.* Plasma inflammatory cytokines and chemokines in severe acute respiratory syndrome. Clin Exp Immunol 136, 95–103 (2004). 15030519 10.1111/j.1365-2249.2004.02415.x PMC1808997

[b6] He L. *et al.* Expression of elevated levels of pro-inflammatory cytokines in SARS-CoV-infected ACE2+ cells in SARS patients: relation to the acute lung injury and pathogenesis of SARS. J Pathol 210, 288–297 (2006). 17031779 10.1002/path.2067 PMC7167655

[b7] Gu J. & Korteweg C. Pathology and pathogenesis of severe acute respiratory syndrome. Am J Pathol 170, 1136–1147 (2007). 17392154 10.2353/ajpath.2007.061088 PMC1829448

[b8] Tse G. M. *et al.* Pulmonary pathological features in coronavirus associated severe acute respiratory syndrome (SARS). J Clin Pathol 57, 260–265 (2004). 14990596 10.1136/jcp.2003.013276 PMC1770245

[b9] Lindner H. A. *et al.* The papain-like protease from the severe acute respiratory syndrome coronavirus is a deubiquitinating enzyme. J Virol 79, 15199–15208 (2005). 16306591 10.1128/JVI.79.24.15199-15208.2005 PMC1316033

[b10] Frieman M., Ratia K., Johnston R. E., Mesecar A. D. & Baric R. S. Severe acute respiratory syndrome coronavirus papain-like protease ubiquitin-like domain and catalytic domain regulate antagonism of IRF3 and NF-kappaB signaling. J Virol 83, 6689–6705 (2009). 19369340 10.1128/JVI.02220-08 PMC2698564

[b11] Chen X. *et al.* SARS coronavirus papain-like protease inhibits the type I interferon signaling pathway through interaction with the STING-TRAF3-TBK1 complex. Protein Cell 5, 369–81 (2014). 24622840 10.1007/s13238-014-0026-3 PMC3996160

[b12] Li S. W. *et al.* Severe acute respiratory syndrome coronavirus papain-like protease suppressed alpha interferon-induced responses through downregulation of extracellular signal-regulated kinase 1-mediated signalling pathways. J Gen Virol 92, 1127–1140 (2011). 21270289 10.1099/vir.0.028936-0

[b13] Li S. W. *et al.* Correlation between TGF-beta1 expression and proteomic profiling induced by severe acute respiratory syndrome coronavirus papain-like protease. Proteomics 12, 3193–205 (2012). 22936401 10.1002/pmic.201200225 PMC7168038

[b14] Lee C. H. *et al.* Altered p38 mitogen-activated protein kinase expression in different leukocytes with increment of immunosuppressive mediators in patients with severe acute respiratory syndrome. J Immunol 172, 7841–7847 (2004). 15187168 10.4049/jimmunol.172.12.7841

[b15] Beijing Group of National Research Project for SARS. Dynamic changes in blood cytokine levels as clinical indicators in severe acute respiratory syndrome. Chin Med J (Engl) 116, 1283–1287 (2003). 14527349

[b16] Rogel M. R. *et al.* Vimentin is sufficient and required for wound repair and remodeling in alveolar epithelial cells. FASEB journal 25, 3873–3883 (2011). 21803859 10.1096/fj.10-170795 PMC3205840

[b17] Kim S. I. *et al.* TGF-beta-activated kinase 1 and TAK1-binding protein 1 cooperate to mediate TGF-beta1-induced MKK3-p38 MAPK activation and stimulation of type I collagen. Am J Physiol Renal Physiol 292, F1471–1478 (2007). 17299140 10.1152/ajprenal.00485.2006

[b18] Presser L. D., McRae S. & Waris G. Activation of TGF-β1 promoter by hepatitis C virus-induced AP-1 and Sp1: role of TGF-β1 in hepatic stellate cell activation and invasion. Plos One 8, e56367 (2013). 23437118 10.1371/journal.pone.0056367 PMC3578869

[b19] Frazier W.A. Thrombospondins. Curr Opin Cell Biol 3, 792–9799 (1991). 1718338 10.1016/0955-0674(91)90052-z

[b20] Yu Q. & Stamenkovic I. Cell surface-localized matrix metalloproteinase-9 proteolytically activates TGF-beta and promotes tumor invasion and angiogenesis. Genes Dev 14, 163–176 (2000). 10652271 PMC316345

[b21] Presser L. D., Haskett A. & Waris G. Hepatitis C virus-induced furin and thrombospondin-1 activate TGF-β1: role of TGF-β1 in HCV replication. Virology 412, 284–296 (2011). 21296375 10.1016/j.virol.2010.12.051 PMC3073624

[b22] Moustakas A. & Heldin C. H. Non-Smad TGF-beta signals. J Cell Sci 118, 3573–3584 (2005). 16105881 10.1242/jcs.02554

[b23] Kim S. J., Glick A., Sporn M. B. & Roberts A. B. Characterization of the promoter region of the human transforming growth factor-beta 1 gene. J Biol Chem 264, 402–408 (1989). 2909528

[b24] Weigert C., Brodbeck K., Klopfer K., Häring H. U. & Schleicher E. D. Angiotensin II induces human TGF-beta 1 promoter activation: similarity to hyperglycaemia. Diabetologia 45, 890–898 (2002). 12107734 10.1007/s00125-002-0843-4

[b25] Yang T. C. *et al.* Japanese encephalitis virus down-regulates thioredoxin and induces ROS-mediated ASK1-ERK/p38 MAPK activation in human promonocyte cells. Microbes Infect 12, 643–651 (2010). 20430109 10.1016/j.micinf.2010.04.007

[b26] Nose K. & Ohba M. Functional activation of the egr-1 (early growth response-1) gene by hydrogen peroxide. Biochem J 316, 381–383 (1996). 8687376 10.1042/bj3160381 PMC1217360

[b27] Hayashi H., Sakai K., Baba H. & Sakai T. Thrombospondin-1 is a novel negative regulator of liver regeneration after partial hepatectomy through transforming growth factor-beta1 activation in mice. Hepatology 55, 1562–1573 (2012). 22105716 10.1002/hep.24800 PMC3295913

[b28] Zhao X., Nicholls J. M. & Chen Y. G. Severe acute respiratory syndrome-associated coronavirus nucleocapsid protein interacts with Smad3 and modulates transforming growth factor-beta signaling. J Biol Chem 283, 3272–3280 (2008). 18055455 10.1074/jbc.M708033200 PMC8740907

[b29] Bhattacharyya S., Fang F., Tourtellotte W. & Varga J. Egr-1: new conductor for the tissue repair orchestra directs harmony (regeneration) or cacophony (fibrosis). J Pathol 229, 286–297 (2013). 23132749 10.1002/path.4131 PMC3965177

[b30] Bhattacharyya S. *et al.* Early growth response transcription factors: key mediators of fibrosis and novel targets for anti-fibrotic therapy. Matrix Biol 30, 235–242 (2011). 21511034 10.1016/j.matbio.2011.03.005 PMC3135176

[b31] Pagel J. I. & Deindl E. Disease progression mediated by egr-1 associated signaling in response to oxidative stress. Int J Mol Sci 13, 13104–13117 (2012). 23202940 10.3390/ijms131013104 PMC3497314

[b32] Hasan R. N. & Schafer A. I. Hemin upregulates Egr-1 expression in vascular smooth muscle cells via reactive oxygen species ERK-1/2-Elk-1 and NF-kappaB. Circ Res 102, 42–50 (2008). 17967787 10.1161/CIRCRESAHA.107.155143

[b33] Son Y. *et al.* Mitogen-Activated Protein Kinases and Reactive Oxygen Species: How Can ROS Activate MAPK Pathways? J Signal Transduct 2011, 792639 (2011). 21637379 10.1155/2011/792639 PMC3100083

[b34] Meng A., Zhang X. & Shi Y. Role of p38 MAPK and STAT3 in lipopolysaccharide-stimulated mouse alveolar macrophages. Exp Ther Med 8, 1772–1776 (2014) 25371731 10.3892/etm.2014.2023 PMC4218692

