# Supplementary material for: SARS coronavirus papain-like protease induces Egr-1-dependent up-regulation of TGF-β1 via ROS/p38 MAPK/STAT3 pathway
Source: Sci Rep. 2016 May 13;6:25754. doi: 10.1038/srep25754 (PMC4865725; doi:10.1038/srep25754)
Supplement: Supplementary Information [file srep25754-s1.pdf]

Supporting Information

SARS coronavirus papain-like protease induces Egr-1-dependent up-regulation of TGF-β1 via ROS/p38 MAPK/STAT3 pathway

Shih-Wein Li[1] Ching-Ying Wang[1] Yu-Jen Jou[1] Tsuey-Ching Yang[2]

Su-Hua Huang[3] Lei Wan[4] Ying-Ju Lin[4] Cheng-Wen Lin [1,3 *]

[1]Department of Medical Laboratory Science and Biotechnology, China Medical University, Taichung, Taiwan
[2]Department of Biotechnology and Laboratory Science in Medicine, National Yang Ming University, Taipei, Taiwan
[3]Department of Biotechnology, Asia University, Wufeng, Taichung, Taiwan
[4]Department of Medical Genetics and Medical Research, China Medical University Hospital, Taichung, Taiwan

*Corresponding author: Cheng-Wen Lin, PhD, Professor. Department of Medical Laboratory Science and Biotechnology, China Medical University, No. 91, Hsueh-Shih Road, Taichung 404, Taiwan, Republic of China

Fax : 886-4-22057414

Email : cwlin@mail.cmu.edu.tw

Beside A549 cells, three other cell lines Huh7 (human hepatocarcinoma), H1299 (human non-small cell lung carcinoma), and ca9-22 (human oral cancer) were used as the control for examining the TGF-β1 induction of SARS-CoV PLpro in different cell lines (Supplemental Fig. 1). Real-time RT PCR analysis of transfected cells with pSARS-PLpro indicated that a lower level of TGF-β1 mRNA was detected in transfected H1299 cells compared to transfected A549 cells, but no significant level was found in transfected Huh7 and ca9-22 cells.

To examine with the mRNA levels of PLpro and TGF-β1 in the transfected cells with virus infected cell**s,** the non-infectious SARS-CoV replicon pBAC-SARSCoV△ES was transfected into A549 cells to mimic the infected cells. Comparison of the expression levels of PLpro and TGF-β1 among transfected cells with empty vector, pSARS-PLpro, and pBAC-SARSCoV△ES (a non-infectious SARS-CoV replicon) was further performed (Supplemental Fig. 2). The expression level of PLpro in transfected cells with pSARS-PLpro was 25-fold higher than the cells transfected with pBAC-SARSCoV△ES. A dose-dependent increase of TGF-β1 mRNA levels in A549 cells was induced by pSARS-PLpro and pBAC-SARSCoV△ES, respectively.

To test the specificity of SARS-CoV PLpro on TGF-β1 induction, MER-CoV and HCoV NL63 PLpro genes were cloned into pcDNA3.1 and then measured their ability of TGF-β1 induction (Supplemental Fig. 3). Interestingly, only SARS-CoV PLpro, but not ERS-CoV and HCoV NL63 PLpro, dose-dependently up-regulated the mRNA expression of TGF-β1.

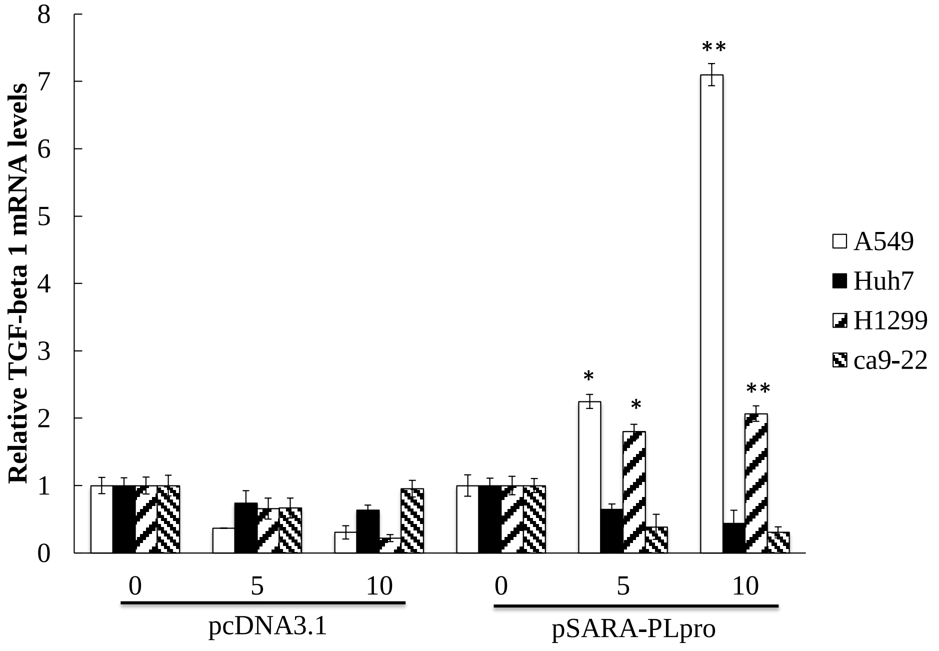

Supplemental Fig. 1. Relative mRNA levels of TGF-β1 in transfected different cell lines with vector control and pSARS-PLpro. A549 (human alveolar basal epithelial), Huh7 (human hepatocarcinoma), H1299 (human non-small cell lung carcinoma), and ca9-22 (human oral cancer) cells were transiently transfected with pcDNA3.1 or pSARS-PLpro. Total RNAs of transfected cells were extracted 1-2 days after transfection. Relative TGF-β1 mRNA level was measured by quantitative real-time PCR, normalized by GAPDH mRNA, and then presented as the relative ratio. *, p value < 0.05; **, p value < 0.01 compared with vector control cells.

A.

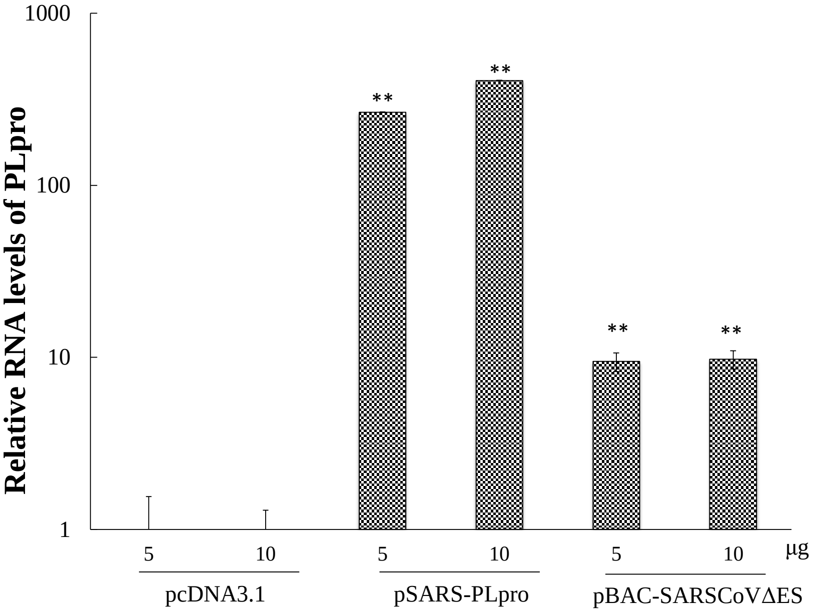

B.

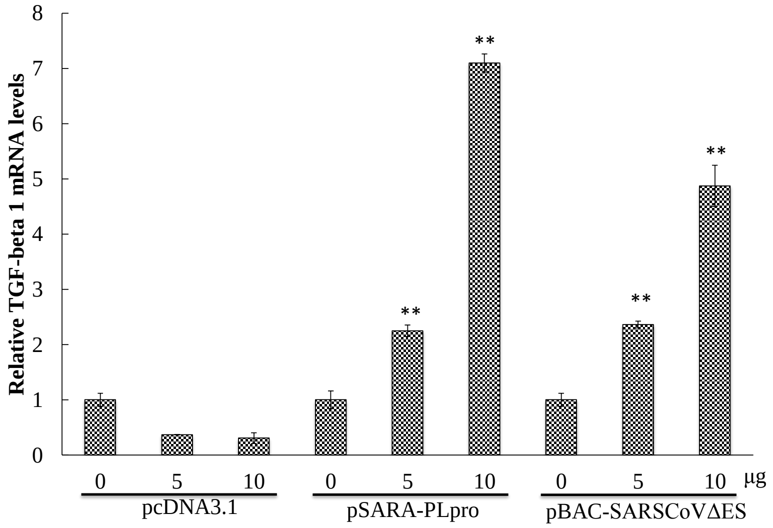

Supplemental Fig. 2. Comparison of SARS-CoV PLpro and TGF-β1 expression levels in A549 cells transfected with pcDNA3.1, pSARS-PLpro, pSARS-PLpro, or pBAC-SARSCoV△ES. The non-infectious SARS-CoV replicon pBAC-SARSCoV△ES was kindly provided by Drs. Zheng-Li Shi and Luis Enjuanes. A549 cells were transiently transfected with empty vector, pSARS-PLpro, or pBAC-SARSCoV△ES 2 days post transfection, harvested for total RNA extraction. The relative levels of SARS-CoV PLpro (A) and TGF-β1 (B) mRNA level are measured by quantitative real time PCR, normalized by GAPDH mRNA, and then presented as the relative ratio. *, *p* value < 0.05; ***p* value < 0.01 compared with vector control cells.

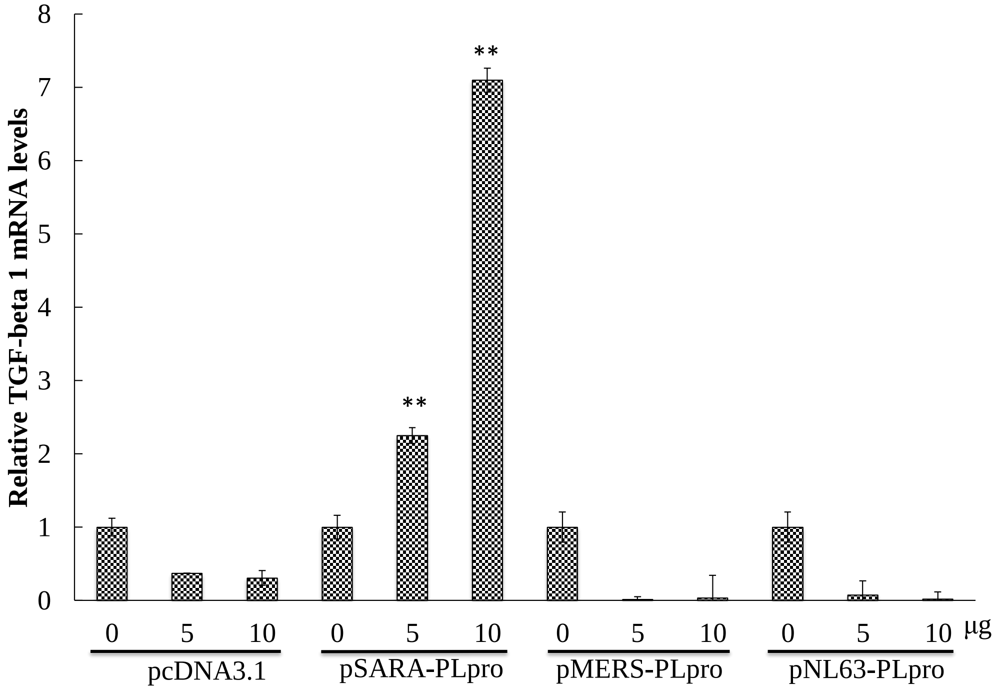

Supplemental Fig. 3. Relative mRNA levels of TGF-β1 in A549 cells transfected with pcDNA3.1, pSARS-PLpro, pMERS-PLpro, and pNL63-PLpro. MERS-CoV and HCoV NL63 PLpro genes were amplified using PCR from the MERS-CoV and HCoV NL63 genome cDNAs that were provided by Dr. Chien-Te K. Tseng and Dr. Lia van der Hoek, respectively. PCR products were cloned into the vector pcDNA3.1. Total RNAs of indicated transfected cells were extracted 1-2 days after transfection. Relative TGF-β1 mRNA level was measured by quantitative real-time PCR, normalized by GAPDH mRNA, and then presented as the relative ratio. **, *p* value < 0.01 compared with vector control cells.
